# C_4_-like photosynthesis and the effects of leaf senescence on C_4_-like physiology in *Sesuvium sesuvioides* (Aizoaceae)

**DOI:** 10.1093/jxb/erz011

**Published:** 2019-01-24

**Authors:** Katharina Bohley, Till Schröder, Jürgen Kesselmeier, Martha Ludwig, Gudrun Kadereit

**Affiliations:** 1 Institut für Molekulare Physiologie, Johannes Gutenberg-Universität, Mainz, Germany; 2 Institut für Organismische und Molekulare Evolutionsbiologie, Johannes Gutenberg-Universität, Mainz, Germany; 3 Philipps-Universität, FB 16–Pharmazie, Marburg, Germany; 4 Max Planck Institute for Chemistry, Multiphase Chemistry Department, Mainz, Germany; 5 School of Molecular Sciences [310], University of Western Australia, Crawley, Western Australia, Australia

**Keywords:** Aizoaceae, carbon isotope values, C_4_-like, C_4_ photosynthesis, immunolocalization of Rubisco and PEPC, portulacelloid leaf anatomy

## Abstract

*Sesuvium sesuvioides* (Sesuvioideae, Aizoaceae) is a perennial, salt-tolerant herb distributed in flats, depressions, or disturbed habitats of southern Africa and the Cape Verdes. Based on carbon isotope values, it is considered a C_4_ species, despite a relatively high ratio of mesophyll to bundle sheath cells (2.7:1) in the portulacelloid leaf anatomy. Using leaf anatomy, immunocytochemistry, gas exchange measurements, and enzyme activity assays, we sought to identify the biochemical subtype of C_4_ photosynthesis used by *S. sesuvioides* and to explore the anatomical, physiological, and biochemical traits of young, mature, and senescing leaves, with the aim to elucidate the plasticity and possible limitations of the photosynthetic efficiency in this species. Assays indicated that *S. sesuvioides* employs the NADP-malic enzyme as the major decarboxylating enzyme. The activity of C_4_ enzymes, however, declined as leaves aged, and the proportion of water storage tissue increased while air space decreased. These changes suggest a functional shift from photosynthesis to water storage in older leaves. Interestingly, *S. sesuvioides* demonstrated CO_2_ compensation points ranging between C_4_ and C_3_–C_4_ intermediate values, and immunocytochemistry revealed labeling of the Rubisco large subunit in mesophyll cells. We hypothesize that *S. sesuvioides* represents a young C_4_ lineage with C_4_-like photosynthesis in which C_3_ and C_4_ cycles are running simultaneously in the mesophyll.

## Teaser

*Sesuvium sesuvioides* represents a young C_4_ lineage with C_4_-like metabolism: CO_2_ compensation points range between C_4_ and C_3_–C_4_ intermediate values, and Rubisco was detected in bundle sheath and mesophyll.

## Introduction

The C_4_ pathway is a carbon-concentrating mechanism which enhances photosynthetic efficiency by concentrating CO_2_ at the site of Rubisco, thereby promoting carbon fixation and reducing photorespiration (Hatch, 1987). To function efficiently, C_4_ photosynthesis requires the controlled separation and close integration of two pathways: (i) phospho*enol*pyruvate (PEP) carboxylation and regeneration and (ii) the Calvin cycle (Bräutigam and Weber, 2011). In most C_4_ species, a spatial separation of these processes is realized: carbon assimilation takes place in mesophyll (M) cells, where PEP carboxylase (PEPC) catalyzes the carboxylation of PEP, while Rubisco and the other Calvin cycle enzymes are localized to the bundle sheath (BS) cells (Dengler *et al.*, 1985). M and BS cells are tightly connected, and a M to BS ratio approaching 1:1 is considered ideal to maximize the efficiency of the C_4_ pathway (Edwards and Voznesenskaya, 2011; Christin and Osborne, 2014). Substantial and complex genetic and structural changes occurred during the evolution of C_4_ plants from their C_3_ ancestors (Sage, 2004). Nevertheless, C_4_ photosynthesis evolved >60 times in land plants, both in monocots and in eudicots (Sage, 2016). Major study systems to unravel the origin and function of C_4_ photosynthesis include *Zea mays* L. (Poaceae), *Flaveria* Juss. (Asteraceae; see Covshoff *et al.*, 2014 for references), and several others (see ‘The C_4_ photosynthesis hall of fame’ list in Sage, 2016; Table 4).

A high diversity of C_4_ leaf anatomy exists in the various C_4_ lineages of sedges, grasses, and eudicots, and has been comprehensively described by Edwards and Voznesenskaya (2011). In eudicots, the most important characteristics to distinguish the different anatomical subtypes are the arrangements of M and BS cells, the arrangement of vasculature and water storage tissue (WST), as well as the presence or absence of hypodermal cells. While most C_4_ species utilize the two-cell system with tightly connected M and BS cells, examples of single-cell C_4_ metabolism have been described (Voznesenskaya *et al.*, 2001).

While many species in the derived subfamilies of Aizoaceae are known to use C_3_/Crassulacean acid metabolism (CAM) (Hartmann, 1993; Klak *et al.*, 2017), the majority of species in the basal subfamily Sesuvioideae employ the C_4_ pathway. To date, four types of C_4_ leaf anatomy (atriplicoid, pilosoid, portulacelloid, and salsoloid) and the C_4_ biochemical subtypes NAD-malic enzyme (NAD-ME) and NADP-malic enzyme (NADP-ME) have been observed in the subfamily (Muhaidat *et al.*, 2007; Bohley *et al.*, 2015; Koteyeva *et al.*, 2015). Taken together, the molecular phylogeny and the evidence of anatomical and biochemical diversity of C_4_ types suggest at least six independent origins of C_4_ in Sesuvioideae (Bohley *et al.*, 2015). Two of these potential C_4_ origins are found in *Sesuvium*, which comprises an African C_4_ clade and an American clade with C_3_ as well as C_4_ species. While the biochemical C_4_ subtype has been determined only for *Sesuvium humifusum* (NADP-ME), which shows atriplicoid leaf anatomy (Muhaidat *et al.*, 2007), previous work has also revealed the presence of pilosoid and portulacelloid leaf anatomy in the genus (Muhaidat *et al.*, 2007; Bohley *et al.*, 2015).

The ontogenetic development of the C_4_ pathway has been studied in few lineages (Muhaidat *et al.*, 2018, and references therein). In monocots, it has been investigated in a number of Cyperaceae and Poaceae species with different leaf types (Nelson, 2011, and references therein). In eudicots, species with the atriplicoid leaf anatomy have received most attention (e.g. *Atriplex rosea*, Dengler *et al.*, 1995; *Amaranthus hypochondriacus*, Wang *et al.*, 1992), whereas only few studies have focused on succulent leaf types. Koteyeva *et al*. (2011, 2014) conducted detailed studies comparing the differentiation of C_4_ anatomy from leaf initiation to full maturation in two species of *Suaeda* Forssk. ex J.F. Gmel. and two species of *Cleome* L., respectively. They found that, despite possessing different leaf anatomies (salsinoid, schoberiod, atriplicoid, and glossocardiod), all four species showed C_4_ cell-specific enzyme expression, although noticeable Rubisco labeling was detected in M chloroplasts of *S. eltonica* Iljin in the youngest leaf stage. In *Cleome gynandra*, a shift in the composition of decarboxylating enzymes is seen, with NAD-ME as the main decarboxylase in young leaves and a combination of NAD-ME and PEPCK used in adult leaves (Sommer *et al.*, 2012). While leaf development in these C_4_*Cleome* and *Suaeda* species does not start from a default C_3_ state, it does so in *Amaranthus hypochondriacus* (Wang *et al.*, 1992), which switches from a C_3_ gene expression pattern at the start of leaf development to NAD-ME-type C_4_ within 24–36 h.

Variation in the temporal development of C_4_ photosynthesis at the whole-plant level is found in some Chenopodiaceae lineages, where cotyledons use C_3_ photosynthesis and foliage leaves employ C_4_. Lauterbach *et al.* (2017) showed that cotyledons of *Salsola soda* L. perform C_3_ metabolism, whereas transcripts encoding the C_4_ enzyme machinery are detectable in the first foliage leaf pair. A possible explanation might be the lower costs of C_3_ metabolism under the growing conditions during germination and seedling stage in *S. soda*. The same pattern is found in other species of the tribe Salsoleae (Pyankov *et al.*, 2001; Li *et al.*, 2015).

Whereas many ontogenetic studies focus on the development of Kranz anatomy and establishment of C_4_ metabolism in developing leaves, the effect of leaf senescence on C_4_ photosynthesis has received much less attention. Imai and Murata (1979) compared photosynthetic properties of mature and senescent leaves from C_3_ and C_4_ Poaceae and Cyperaceae species. They found a general decline in photosynthetic activity in all species as leaves aged, as demonstrated by decreasing apparent photosynthetic rates and increasing CO_2_ compensation points (Γ). In the C_4_ eudicot *Portulaca oleracea* L., a shift occurs from low levels of C_3_ primary photosynthetic products in young and mature leaves towards higher levels in senescent leaves, indicating an increased involvement of the C_3_ cycle in the fixation of atmospheric CO_2_ (Kennedy and Laetsch, 1973). The authors suggested that, in this species, C_4_ photosynthesis might be a complementary carbon assimilation mechanism during certain ontogenetic stages, albeit potential age-related effects were not discussed.

The current study focuses on *S. sesuvioides* (Fenzl) Verdc., a perennial herbaceous C_4_ species from southern Africa. Our previous work (Bohley *et al.*, 2015) showed that this species demonstrates portulacelloid leaf anatomy, where BS and M surround each vein and the chlorenchyma is arranged at the adaxial side of the leaf. High amounts of WST in combination with relatively small BS cells and a high M:BS area ratio led us to question whether the efficiency of the C_4_ pathway in this species is affected by anatomical constraints. For the current study, 17 individuals of *S. sesuvioides* were cultivated in the greenhouse for 2 years, with their C_4_ performance examined during aging. We used immunocytochemical techniques in combination with gas exchange measurements and enzyme activity assays to assess potential differences in performance between leaves of different ages within individuals and also among individuals of different ages. Specific questions we aimed to answer were: what is the biochemical C_4_ subtype of *S. sesuvioides* and how does this relate to its closest C_4_ relatives in Sesuvioideae? Do the high M:BS ratio and the changing anatomy in aging leaves influence the C_4_ characteristics and photosynthetic efficiency of *S. sesuvioides*?

## Materials and methods

### Plant material

Plants were grown from seeds collected at two locations, South Africa and Namibia. The population from this point on referred to as ‘wild-1’ was collected at Richtersveld Nursery, Northern Cape, South Africa [~28.1240250°S, 16.8918139°E; voucher: A. J. Moore s. n. (MJG)]. The second population (wild-2) was collected ~80 km east of Sendelingsdrif, Karas, Namibia [~28.20946°S, 17.28936°E; 208 m altitude; voucher: Klak 2431 (BOL)]. Seeds were germinated in Petri dishes on moist activated charcoal filter paper. After germination, the seedlings were planted in a mixture of peat and sand, and were transferred to a potting mix of 60% soil of volcanic origin (pumice and lava), 20% compost, and 20% fine gravel at an age of ~8 weeks. The plants were watered as needed and fertilized once a week with 0.1% Wuxal Super (Manna, Willhelm Haug GmbH & Co. KG, Düsseldorf, Germany). The plants were kept in a greenhouse at a minimum temperature of 18 °C in the night. Daytime temperatures usually reached values of 25–35 °C in the summer months and 20–25 °C in the winter months. Day/night cycles of 14 h/10 h were maintained with natural illumination and supplementary light (usually >400 µmol m^−2^ s^−1^; sometimes at minimum intensity of ~200 µmol m^−2^ s^−1^ at plant level, e.g. during dense cloud cover during winter).

### Age stages of greenhouse-grown plants

During the measurements (see below), it became apparent that plants from both source populations behaved similarly. We therefore decided not to differentiate between these source populations. Seventeen plants were used throughout the study and were each individually identified by a lower case letter (a–q; see Table 1). The leaves along a branch were divided into three stages starting from the tip: young leaves (y in tables and figures) were leaves from the second or third node and had nearly reached full expansion. Mature leaves were from node five or six, had reached full expansion, and were considered to be photosynthetically fully active. Senescing leaves were harvested from older parts of the branches (seventh node or older) and were chosen by eye based on increased succulence in combination with paler coloration compared with mature leaves. Leaves at this stage were not immediately abscised by the plant, but rather stayed in this stage for time spans of several weeks or even months. It has to be noted that not all individuals provided leaves of this last age category.

**Table 1.** Germination date, stable carbon isotope values relative to the Pee Dee belemnite standard (*n*=1 for all samples with three technical replicates), and mean CO_2_ compensation points of *Sesuvium sesuvioides* individuals cultivated in the greenhouse

		Carbon isotope measurements	Gas exchange measurements
	Germination	δ^13^C value ±SD (‰)^*a*^	Γ_mean_ ±SD (ppm)
wild-1	NA	–14.013±0.071	NA
wild-2	NA	–14.451±0.081	NA
a	01/2016	–14.228±0.065	0.97±2.34
b	01/2016	–15.521±0.008	2.91±1.69
c	09–10/2015	–15.088±0.066	10.17±10.45
d	09–10/2015	–15.122±0.060	6.41±2.43
e	09–10/2015	–15.181±0.049	3.17±3.23
f	09–10/2015	–15.607±0.039	5.08±1.89
g	09–10/2015	–15.641±0.020	3.78±2.59
h	09–10/2015	–15.683±0.172	5.93±4.26
i	09–10/2015	–15.667±0.074	3.36±2.18
j	09–10/2015	–16.041±0.055	6.63±3.86
k	03–04/2015	–15.213±0.029	11.12±5.92
l	03–04/2015	–15.661±0.060	6.41±2.64
m	03–04/2015	–15.904±0.049	9.18±1.48
n	03–04/2015	–16.176±0.015	5.12±4.55
o	03–04/2015	–16.356±0.026	8.44±3.69
p	03–04/2015	–16.802±0.026	7.88±2.28
q	03–04/2015	–16.885±0.021	8.39±2.84
*Salsola verticillata* (C_3_), young plants (*n*=2)^*b*^		–31.39	NA
*Salsola oppositifolia* (C_4_), young plant^*b*^		–17.51	NA
*Salsola soda* (C_4_), adult plant^*b*^		–15.87	NA

Stable carbon isotope values of the wild source plants are also shown; however, no data on germination and gas exchange measurements of these are available (NA). Detailed information about the gas exchange measurements can be found in Supplementary Table S2. For comparison, isotope values of a C_3_ and a C_4_ species grown in the same greenhouse are included

a All samples harvested at the same time (12 September 2016).

b Unpublished data.

c Lauterbach *et al.*, (2017)

In addition to the described categories, cotyledons and leaves of young seedlings (3 weeks old) were also used for sectioning and subsequent immunolabeling. Besides the cotyledons, seedlings at this age usually had the young first leaf pair and sometimes an emerging second leaf pair (≤1 mm), of which the former two were harvested simultaneously. To account for potential changes with increasing leaf age, cotyledons and leaves of 6-week-old seedlings were also sampled for immunolabeling (cotyledons, and first and second leaf pairs). No other measurements were conducted with any of the seedlings due to their small size.

### Leaf isotope measurements

For carbon isotope measurements, mature leaves of plants in the greenhouse were harvested in September 2016 and dried with silica gel. These samples and additional material from the wild populations, as well as samples of other C_3_ and C_4_ species grown in the same greenhouse, were ground to fine powder with a mixer mill MM301 (Retsch, Haan, Germany). Stable carbon isotope values relative to the Pee Dee belemnite standard were determined from ~200 µg of sample with the stable isotope ratio mass spectrometer MAT253 (Thermo Scientific, Wilmington, DE, USA), an organic elemental analyzer Flash 2000 Elemental Analyzer (Thermo Scientific), and a Conflo IV (Thermo Scientific) at the Institut für Geowissenschaften (Institute for Geological Sciences) at Johannes Gutenberg-Universität, Mainz, Germany. Three technical replicates were analyzed for each sample to ensure the accuracy of results.

### Gas exchange measurements

Whole-leaf gas exchange was measured under ambient conditions in the greenhouse using a GFS-3000 (WALZ, Germany). Due to the construction of the cuvette (20 mm×40 mm×20 mm) and the morphology of the species, which has dense foliage, a mix of young and mature leaves were enclosed and contributed to the results. In all measurements, the distal parts of branches with usually ~10 leaves (range 5–15) were used. We decided against removing leaves to minimize stress-related reactions of the plants.

The leaves were illuminated with a light intensity of 1400 µmol m^−2^ s^−1^ photosynthetic photon flux density (PPFD), and were allowed to acclimatize to these light conditions at 380 ppm CO_2_, 25 °C prior to the beginning of the measurements. The minimum time for acclimatization was 10 min in combination with a stability criterion of the GFS-3000, which prevents the machine from executing the next step in the measurement regime unless stable measured values are detected. The relative humidity in the cuvette was set to 42%, but usually reached levels between 45% and 60% after acclimatization. The measurements started with a CO_2_ concentration of 380 ppm, which was lowered stepwise to a concentration of 0 ppm, followed by steps to 380 ppm and 750 ppm. Furthermore, we tested if CO_2_ uptake could be detected after the end of the light period to determine if CAM activity is present in the species. The settings for these measurements followed a 14 h light/10 h dark rhythm with 380 ppm CO_2_, 25 °C, and 42% relative humidity inside the cuvette, and an illumination of 1400 µmol m^−2^ s^−1^ PPFD during the light phase (06.00–20.00 h) and 0 µmol m^−2^ s^−1^ PPFD during the dark phase (20.00–06.00 h). Calibrations of the analyzer were conducted as suggested by the manufacturer, with calibration gas standards for CO_2_ (Air Liquide, Paris, France) and a dew point generator (Li 610; LICOR, Lincoln, NE, USA) for the calibration of water vapor.

The compensation point (Γ) was calculated from the *x*-intercept of the *A*/*C*_i_ curve at the five lowest levels of ambient CO_2_.

### Titratable acidity

To investigate further possible CAM activity in *S. sesuvioides*, titratable acidity was compared for leaves harvested at the beginning and the end of the light period (05.35–05.55 h and 19.45–20.03 h, respectively). Mature leaves of the individuals a, b, d, k, l, n, o, and p were harvested, weighed, and snap-frozen in liquid nitrogen. For each time point, leaves from different branches within each individual were sampled, and one replicate per individual and time point was included in the analysis. The leaf material was finely chopped and boiled in 20% ethanol for 60 min. After cooling to room temperature, each extract was aliquoted into triplicates of equal volume that were titrated to neutrality by adding 0.1 M NaOH in 1 µl or 2 µl increments (Christin *et al.*, 2014).

### Enzyme activity assays

The activity of PEPC was measured according to Boyd *et al.* (2015). NAD-ME and NADP-ME activities were measured following the protocols provided in Muhaidat *et al.* (2007). The enzyme activities were recorded relative to the chlorophyll content, which was extracted in a standard volume of 1 ml of 96% ethanol following the protocol of Wintermans and de Motts (1965). The protocols were modified for use with a 96-well plate reader (infinite M1000, Tecan, Austria). To account for possible pipetting errors, each sample was measured in triplicate. For each individual, leaves of different age stages, as described above, were sampled.

For comparison, enzyme activities of *Salsola oppositifolia* Desf. (C_4_) and *Sesuvium verrucosum* Raf. (C_3_) are provided in Table 3. *Sesuvium verrucosum* was cultivated in the same greenhouse as *S. sesuvioides*, while *S. oppositifolia* was cultivated in a nearby greenhouse. The assays of *S. verrucosum* and *S. oppositifolia* were done using the same protocols and assay conditions as those with *S. sesuvioides*.

### Leaf anatomy and immunocytochemistry

Leaf material of the different leaf stages was prepared for sectioning and immunolocalization from a subset of the 17 individuals used in this study. As described above, some cotyledons and subsequent leaves from young seedlings were also used for these labeling studies. Immediately after harvesting, leaf/cotyledon samples were fixed overnight with 2% (v/v) glutaraldehyde and 2% (w/v) freshly made paraformaldehyde in 0.1 M phosphate buffer, pH 7.2 (Voznesenskaya *et al.*, 1999). The material was dehydrated in a graded ethanol series and subsequently infiltrated with the methacrylate resin Technovit 7100 as described in the manufacturer’s manual (Heraeus Kulzer; for more details, see the online supplementary material for Bohley *et al.*, 2015). Embedding and curing were done at room temperature. Sections were cut at a thickness of 3–9 µm with a rotary microtome (Leitz, Germany).

Sections were stained with a mixture of Azur II [0.04% (w/v) in distilled water; Merck AG, Germany], methylene blue [0.3% (w/v) in 25% ethanol; Merck AG, Germany], and Eosin Y [0.08% (w/v) in distilled water; Merck AG, Germany] in a ratio of 9:2:3. Images of the sections were collected with a Diaplan microscope (Leitz, Germany) using a Leica DFC 420 C digital camera (Leica, Germany) and the Leica Application Suite V.3.8.0 [Build: 878]. Subsequent analyses were done using Fiji with ImageJ 1.51j (Schindelin *et al.*, 2012; Schneider *et al.*, 2012).

For comparison, the proportions of WST, airspace, M, and BS in relation to the area of a section excluding the epidermis were calculated for the three leaf stages (young, nine individuals; mature, eight individuals; and senescing, eight individuals).

Immunolabeling was done using DAKO EnVision^®^+ System-HRP (AEC) (Agilent, USA) according to the manufacturer’s protocol, apart from the following changes. Sections were initially treated with permeabilization buffer [0.2% (w/v) Triton X-100 in 1× DPBS (Dulbecco’s phosphate-buffered saline), prepared from 10× DPBS (SAFC, USA)] for 20 min to enhance their wettability. The incubation of the endogenous peroxidase blocking step was extended to 20 min, and a 60 min incubation with blocking buffer (3% BSA in DPBS) was added prior to labeling to prevent non-specific binding of primary antibodies. For detection of Rubisco and PEPC, sections were incubated overnight at 4 °C in a moisture chamber with the following dilutions of primary antibody: 1:250 rabbit anti-PEPC antibody (Rockland, USA) or 1:200 rabbit anti-Rubisco large subunit (RbcL) antibody (Bioss, USA). Dilutions were prepared with antibody dilution buffer (1% BSA in DPBS). The polymer-conjugated secondary antibody was diluted 1:5 with HPLC gradient grade water (ROTISOLV, Carl Roth GmbH, Germany) and the incubation time was extended to 60 min. Incubation with the chromogen was also extended to 60–90 min. Sections were counterstained in 1% (w/v) Azur II in double-distilled water (Carl Roth GmbH, Germany) for 40 s. Substitution of the diluted primary antibody with antibody dilution buffer served as the labeling control (Supplementary Fig. S2 at *JXB* online).

### Statistical analyses

All statistical analyses were conducted in R (R Core Team, 2018). The influence of leaf age on anatomical and physiological properties was tested for significance by using the lme function of the package nlme (Pinheiro *et al.*, 2018) to construct linear mixed effects models fitted by restricted maximum likelihood. Model selection was decided based on the Bayesian information criterion (BIC; Schwarz, 1978). In case significant effects could be observed, we used the glht function of the package multcomp (Hothorn *et al.*, 2008) to conduct Holm–Bonferroni-corrected multiple pairwise comparisons.

For testing the relationships between leaf age and enzyme activities (PEPC, NADP-ME, and NAD-ME), chlorophyll content, and leaf anatomy, leaf age was entered as a fixed effect and individual identity as a random effect in the respective models. Since only one fixed effect (leaf age) was defined in the full models, the respective reduced models used for comparison were intercept-only models (estimating the mean of the data), with individual identity as random effect.

Prior to the analyses, the data sets were examined for violations against the assumptions of linear mixed effects models, and residual plots were checked to ensure homoscedasticity before utilizing the model results. Because the visual inspection of residual plots indicated heteroscedasticity in the data sets for BS percentage in a leaf and chlorophyll content, a transformation employing Tukey’s ladder of Power was performed on these data sets. The appropriate λ for this power transformation was determined using the function transformTukey of the package rcompanion (Mangiafico, 2018), which resulted in λ= –0.3 for the data in BS percentages and in λ= –0.325 for the data on chlorophyll content. The transformed data sets were subjected to the same tests as the untransformed sets prior to utilizing model results.

To test the significance of the differences between titratable acidities of samples taken at the end of the light period with those taken at the end of the dark period, the Wilcoxon Signed Rank Test was used.

## Results

### Gas exchange and stable carbon isotope measurements

To determine the CO_2_ compensation point of *S. sesuvioides* individuals, the response of assimilation to decreasing ambient CO_2_ concentrations was measured. Mean compensation points ranged from 1.52 ppm to 10.61 ppm (Table 1; Supplementary Table S1). While this may be attributed to the composition of leaf ages inside the cuvette during each measurement, it may also reflect high variation in Γ in *S. sesuvioides*.

Stable carbon isotope (δ^13^C) values ranged from –14.23‰ to –16.89‰ for the *S. sesuvioides* individuals grown in the greenhouse (Table 1). Values for the leaf material of the source plants from the collection site (wild-1 and wild-2) were –14.01‰ and –14.45‰, respectively (Table 1). There may be a subtle trend of more negative carbon isotope values in older individuals (Table 1); however, to examine plant age-related effects properly, a larger and more balanced sample size would be necessary.

### Leaf anatomy and immunolocalization

**Fig. 1.**
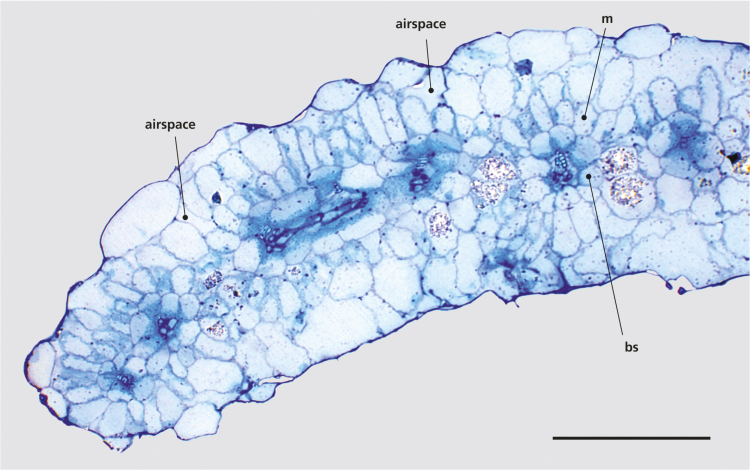
Transverse section through a 3-week-old cotyledon of *Sesuvium sesuvioides*. The cotyledon has an atriplicoid anatomy with a single layer of mesophyll (M) surrounding the bundle sheaths (BS). Intercellular airspace is present on both sides of the leaf. Scale bar=300 µm.

The cotyledons of *S. sesuvioides* had atriplicoid leaf anatomy with a single layer of loosely arranged parenchyma cells and intercellular airspaces on both sides of the leaf (Fig. 1). The chloroplasts in the BS were observed in a centripetal position, while those in the M cells were not clustered. In the first leaf pair, the portulacelloid anatomy of the adult plants was realized (not shown). Vasculature and chlorenchyma were arranged at the adaxial side of the leaf, while stomata were distributed evenly on both leaf surfaces. The BS cells often did not completely encircle the vein, but rather left a gap at the abaxial side where M and vein tissue were in direct contact.

**Fig. 2.**
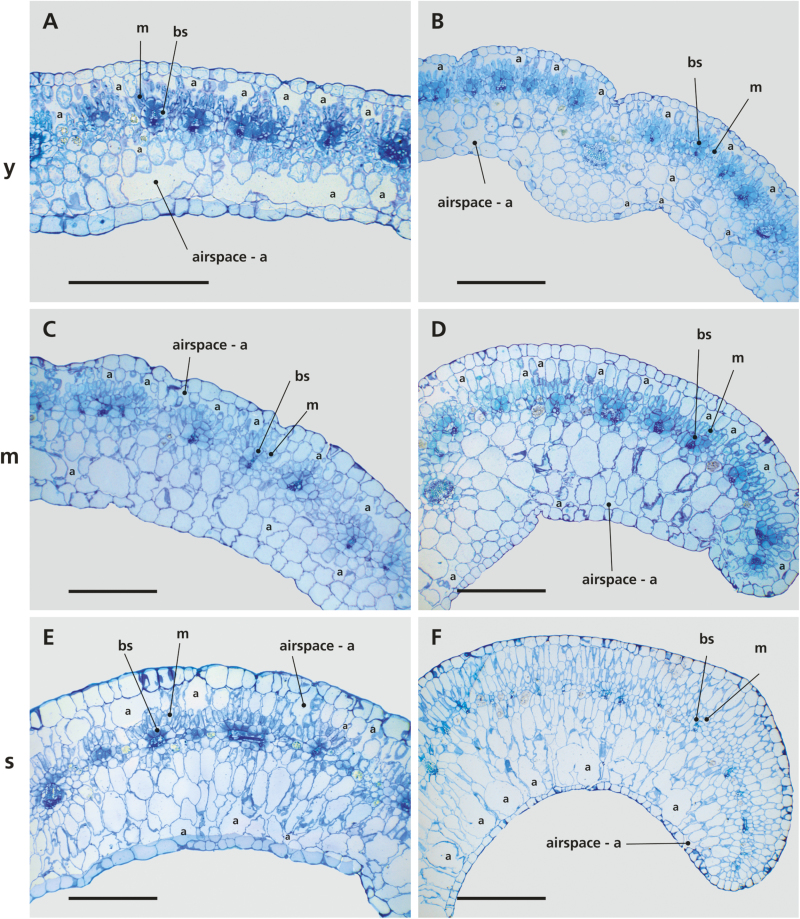
Transverse sections through *Sesuvium sesuvioides* leaves of different ages. (A, C, and E) The leaf anatomy of the youngest individual in the experiment. (B, D, and F) The leaf anatomy of one of the oldest individuals. (A and B) Young leaf (y); (C and D) mature leaf, middle age (m); (E and F) senescing (s) leaf. M, mesophyll; BS, bundle sheath. Scale bars=500 µm.

In young leaves (second or third node from the tip) of mature *S. sesuvioides* individuals, loosely arranged parenchyma and WST cells, along with abundant intercellular airspace, were present on both sides of the chlorenchyma (Fig. 2A, B; Table 2). High numbers of chloroplasts were found clustered in the centripetal position in BS cells, whereas M cells contained lower numbers of evenly dispersed chloroplasts. With increasing leaf age, free airspace decreased (Table 2), while the area occupied by cells, especially on the abaxial leaf surface, appeared to increase (Fig. 2). WST developed with two to several layers on the abaxial and one layer on the adaxial side of the leaf, and accounted for more than two-thirds of a section’s area in senescing leaves (Table 2). We found a M:BS area ratio of 2.7 in young leaves, 3.6 in mature leaves, and 3.7 in senescing leaves.

**Table 2.** Percentage of mesophyll, water storage tissue and airspace in leaves of different ages of *Sesuvium sesuvioides*

Leaf stage	Individuals	Mesophyll ±SD (%)	Bundle sheath ±SD (%)	M:BS ratio	Water storage ±SD (%)	Airspace ±SD (%)
y	a, e, g, h, l, n, o, p, q	28.66±5.49	11.83±3.79	2.71±1.16	45.08±6.89	14.44±3.14
m	a, e, g, h, l, n, o, p	20.95±2.83	6.47±1.89	3.56±1.69	64.68±4.81	7.86±2.85
s	a, e, g, l, n, o, p, q	15.95±4.28	4.58±1.17	3.69±1.51	74.95±5.94	3.66±2.68

The leaf stages are coded as follows: young (y), mature (m), and senescing (s). All percentages and the M:BS ratio are given in relation to the area of a leaf section, not including the epidermis

Linear mixed effects models were used to test the influence of the leaf stage on the percentages of M, BS, WST, and airspace (Table 3). The results of summarizing *F*-statistics indicated a significant influence of the leaf stage on M (*F*-value=27.379, *P*-value ≤0.001), WST (*F*-value=122.561, *P*-value ≤0.001), and airspace (*F*-value=47.039, *P*-value ≤0.001). For the analysis of the influence of leaf stage on the percentage of BS within a leaf, transformed data were used. As was the case for the other leaf compartments, leaf age also had a significant influence on BS (*F*-value=44.1409, *P*-value ≤0.001). For comparison, the same analysis was also done with the non-transformed data to show potential differences in the results (Supplementary Data S1). Multiple comparisons revealed that the differences in airspace, BS, M, and WST were significant between all leaf stages (Supplementary Table S2).

**Table 3.** Results of linear mixed effects models used to assess the effect of leaf stage on the percentage of BS, M, WST, and airspace within an anatomical leaf section

	Value	SE	df	*t*-value
**Bundle sheath** (power-transformed data; λ= –0.3)				
Reduced model^*a*^ (BIC= –39.24976)				
Full model (BIC= –53.27009); *P*-value of fixed effect (leaf age) ≤0.0001				
Leaf_young_	–0.486	0.017	14	5.741
Intercept (Leaf_mature_)	–0.58	0.017	14	–33.464
Leaf_senescing_	–0.638	0.017	14	–3.359
**Mesophyll**				
Reduced model^*a*^ (BIC=173.2147)				
Full model (BIC=147.2821); *P*-value of fixed effect (leaf age) ≤0.0001				
Leaf_young_	28.661	1.753	14	4.274
Intercept (Leaf_mature_)	21.169	1.538	14	13.768
Leaf_senescing_	15.811	1.819	14	–2.946
**Water storage tissue**				
Reduced model^*a*^ (BIC=207.6949)				
Full model (BIC=156.8184); *P*-value of fixed effect (leaf age)≤0.0001				
Leaf_young_	45.079	1.963	14	–9.819
Intercept (Leaf_mature_)	64.36	2.051	14	31.386
Leaf_senescing_	75.19	2.045	14	5.296
**Airspace**				
Reduced model^*a*^ (BIC=161.7314)				
Full model (BIC=129.0645); *P*-value of fixed effect (leaf age) ≤0.0001				
Leaf_young_	14.436	1.134	14	5.755
Intercept (Leaf_mature_)	7.912	1.029	14	7.692
Leaf_senescing_	3.576	1.178	14	–3.683

Model selection was decided based on BICs.

^*a*^ The reduced model is an intercept-only model with individual identity as random effect.

**Fig. 3.**
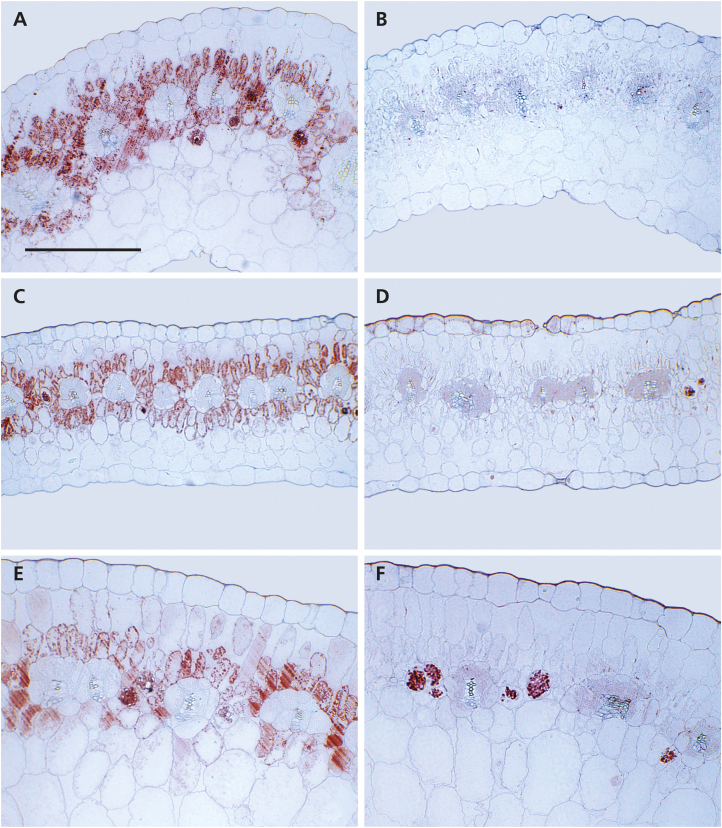
Immunolabeling of phospho*enol*pyruvate carboxylase (PEPC) and Rubisco in transverse sections of different aged leaves of *Sesuvium sesuvioides.* (A, C, and E) PEPC; (B, D, and F) Rubisco. (A and B) Young leaf (y); (C and D) mature leaf, middle age (m); (E and F) senescing (s) leaf. Immunolabeling is represented by the red color in the sections. PEPC labeling is limited to the mesophyll (M) cells in all leaf stages. Labeling of Rubisco is concentrated in the bundle sheaths (BS) in all leaf stages, but was also found to a lesser degree in mesophyll (M) cells. The scale bar in (A) is also valid for (B–F) 300 µm.

**Fig. 4.**
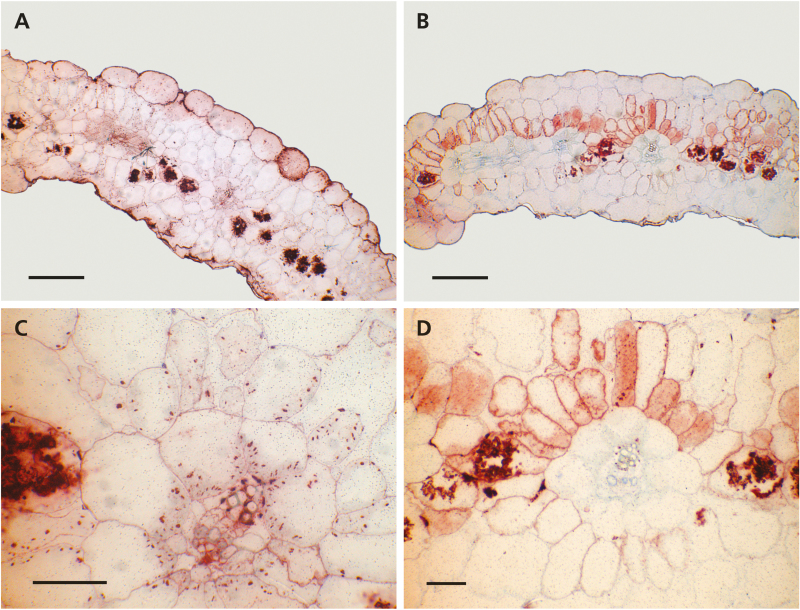
Immunolabeling of transverse sections through a 3-week-old *Sesuvium sesuvioides* cotyledon. (A) Rubisco labeling. (B) Phospho*enol*pyruvate carboxylase (PEPC) labeling. (C) Higher magnification of the Rubisco labeling. Labeled chloroplasts in the mesophyll (M) cells are clearly visible. (D) Higher magnification of the PEPC staining. No label is detected in the bundle sheath (BS) compartment. Scale bars=200 µm in (A and B); 50 µm in (C and D).

The cellular distribution of PEPC and RbcL in leaves of *S. sesuvioides* individuals was investigated using immunolabeling. Labeling of PEPC localized to the M layer directly adjacent to the BS cells and to a lesser degree in peripheral M cells without BS contact (Fig. 3A, C, E). Interestingly, PEPC label was found in all M cells surrounding a BS in young and mature leaves, but was sometimes absent in abaxial M cells in senescing leaves (e.g. Fig. 3E). RbcL labeling was found foremost in the BS compartment (Fig. 3B, D, F). However, a low, but noticeable amount of label was also detected in M cells. In sections labeled for PEPC or RbcL, the precipitated AEC dye of the immunolabeling kit non-specifically bound to calcium oxalate crystals in cells adjacent to M and BS cells caused artifacts (especially visible in the sections labeled for RbcL shown in Figs 3 and 4). These crystal inclusions seem to be common in Sesuvioideae and were found in most sections prepared for the current and previous studies. Immunolabeling of both enzymes was also applied to several cotyledons and the first leaves of young seedlings. In *S. sesuvioides* cotyledons, RbcL labeling was detected in BS and also in M cells (Fig. 4A, C), whereas the anti-PEPC antibody labeled only the M cells on the adaxial side of the leaf (Fig. 4B, D). Cotyledons of 6-week-old seedlings were also labeled (not shown). Although these older cotyledons showed clear signs of senescence, the general labeling pattern for both enzymes was similar to that observed in the 3-week-old cotyledons. The labeling in the first true leaves (not shown) did not differ from that found in young and mature leaves of adult plants.

### Examination of Crassulacean acid metabolism

Eight *S. sesuvioides* individuals (a, b, d, k, l, n, o, and p) were tested for CAM activity by comparing titratable acidity at the end and the beginning of the light period (Supplementary Table S3). No significant difference was detected between the two sampling times (*P*=0.25); therefore, it is unlikely that *S. sesuvioides* uses CAM for CO_2_ assimilation under the growing conditions used in the experiment. Additionally, the measurement of net CO_2_ exchange in 20 h did not indicate CAM activity, but instead showed CO_2_ assimilation during the day and CO_2_ emission during the night (Supplementary Fig. S3).

### Enzyme activity assays and chlorophyll content

The activity of PEPC, NADP-ME, and NAD-ME, as well as chlorophyll content, in three leaf stages were measured and compared (Table 4). PEPC activity was highest in young leaves (146.79±65.56 µkat) and declined towards the senescing leaf stage (mature, 90.12±55.67 µkat; senescing, 63.58±50.24 µkat). The maximum activity of NADP-ME was found in the mature leaf (99.43±49.93 µkat), whereas young leaves (85.87±52.03 µkat), as well as senescing leaves (79.65±65.15 µkat), exhibited slightly lower activities of this enzyme. While the activities of PEPC and NADP-ME reach comparable values in mature leaves, PEPC activity was very high relative to NADP-ME activity in young leaves. In comparison with the activities of NADP-ME, the activity of NAD-ME was low in all three leaf stages (Table 4, column 4). The Chl *a*+*b* content declined from the young leaf stage towards the senescing leaf (Table 4, column 5).

**Table 4.** Activities of C_4_ enzymes and chlorophyll content in *Sesuvium sesuvioides* leaves of different age stages

	Enzyme activity ±SD (µkat)			
Leaf age	PEPC^1^	NADP-ME^1^	NAD-ME^2^	Chl *a*+*b*^1^ (µg L^−1^ mg FW^−1^)
y	146.79±65.56 (*n*=17)	85.87±52.03 (*n*=15)	2.13±15.9 (*n*=6)	27.07±12.72 (*n*=17)
m	90.12±55.67 (*n*=17)	99.43±49.93 (*n*=15)	–0.21±11.08 (*n*=6)	15.37±9.22 (*n*=17)
s	63.58±50.24 (*n*=9)	79.65±65.15 (*n*=8)	5.37±11.08 (*n*=6)	8.04±2.78 (*n*=9)
*Sesuvium verrucosum* (C_3_)	19.99±10.36	6.68±16.76	–0.54±3.25	57.29±6.01
*Salsola oppositifolia* (C_4_)	121.22	74.13±10.67	13.18±14.66	42.67±12.25

The stages are coded as follows: young (y), mature (m), and senescing (s). See the Materials and methods for more details on the assignment of stages. Values of a C_3_ and a C_4_ species are given for comparison. *n* denotes the number of individual measurements (including repeated measures of individuals).

Superscript numbers denote the individuals used in the measurements: ^1^a, d, g, h, j, k, l, n, o, and p; ^2^a, k, l, n, o, and p.

The effect of leaf stage on enzyme activity was tested using linear mixed models. Although the BIC always supported the full model, the leaf stage only had a significant effect on PEPC activity (*F*-value=9.463, *P*-value=0.006) and chlorophyll content (*F*-value=17.619, *P*-value ≤0.001), but not on NADP-ME (*F*-value=0.966, *P*-value=0.393) or NAD-ME activities (*F*-value=0.261, *P*-value=0.776; Table 5). For testing the influence of leaf stage on chlorophyll content, transformed data were used; however, similar calculations with the untransformed data yielded comparable results (Supplementary Data S1). PEPC activity was generally highest in young leaves, and declined with leaf age (Table 4). The differences between young and mature leaves [56.67 µkat±17.93 (SE), *P*-value=0.003], and between young and senescing leaves [87.48 µkat±21.73 (SE), *P*-value ≤0.001] were significant, whereas the difference between mature and senescing leaves was not [30.81 µkat±21.73 (SE), *P*-value=0.156]. On a fresh weight basis, Chl *a+b* content declined from the young leaves to senescing leaves (Table 4). As mentioned above, the results for the chlorophyll content are based on data subjected to a power transformation. Significant differences were found between young and mature leaves [transformed data, –0.075±0.022 (SE), *P*-value=0.001], young and senescing leaves [transformed data, –0.156±0.026 (SE), *P*-value≤0.001], as well as between senescing and mature leaves [transformed data, 0.0763±0.026 (SE), *P*-value 0.004]. The results of a similar analysis using the non-transformed data can be found in Supplementary Data S1.

**Table 5.** Results of linear mixed effects models used to assess the effect of leaf stage on enzyme activities (PEPC, NADP-ME, and NAD-ME) and chlorophyll content

	Value	SE	df	*t*-value
**PEPC**				
Reduced model^*a*^ (BIC=486.73)				
Full model (BIC=463.18); *P*-value of fixed effect (leaf age)=0.0006				
Leaf_young_	143.954	17.926	31	3.161
Intercept (Leaf_mature_)	87.283	15.478	31	5.639
Leaf_senescing_	56.469	21.728	31	–1.418
**NADP-ME**				
Reduced model^*a*^ (BIC=417.6734)				
Full model (BIC=408.043); *P*-value of fixed effect (leaf age)=0.3933				
Leaf_young_	81.627	14.862	27	–0.724
Intercept (Leaf_mature_)	92.384	15.955	27	5.79
Leaf_senescing_	67.352	18.196	27	–1.376
**NAD-ME**				
Reduced model^*a*^ (BIC=153.3626)				
Full model (BIC=145.8305); *P*-value of fixed effect (leaf age)=0.8905				
Leaf_young_	2.129	9.605	10	0.244
Intercept (Leaf_mature_)	-0.21	6.792	10	–0.031
Leaf_senescing_	-2.522	9.605	10	–0.241
**Chlorophyll content** (power-transformed data; λ= –0.325)				
Reduced model^*a*^ (BIC= –67.6178)				
Full model (BIC= –74.2226); *P*-value of fixed effect (leaf age) ≤0.0001				
Leaf_young_	0.429	0.022	31	3.46
Intercept (Leaf_mature_)	0.505	0.017	31	–24.855
Leaf_senescing_	0.582	0.026	31	–2.959

Model selection was decided based on BICs.

a The reduced model is an intercept-only model with individual identity as random effect.

## Discussion

Based on carbon isotope values, *S. sesuvioides* is described as a C_4_ species; however, prior anatomical results revealed a high M:BS area ratio, abundant WST, and relatively small BS cells (Bohley *et al.*, 2015). The current study aimed to gain more information on the physiological and biochemical characteristics of the species and to answer the question of whether its C_4_ capacity is influenced by the anatomical properties. Intriguingly, the results of this study suggest that *S. sesuvioides* may be a C_4_-like species with NADP-ME as its major leaf decarboxylase. Furthermore, the leaves of this species appear to exhibit photosynthetic plasticity as they age, which influences leaf anatomy and PEPC activity.

### *Sesuvium sesuvioides* is an NADP-ME subtype C_4_ plant

The phylogeny of Sesuvioideae suggested either a single origin of C_4_ photosynthesis with reversals to C_3_ photosynthesis, or alternatively at least six independent origins of C_4_ photosynthesis (Bohley *et al.*, 2015). The major decarboxylase used by these C_4_ taxa is only reported for a few species in this subfamily (five out of ~55 C_4_ species have been examined; Supplementary Fig. S1). *Trianthema sheilae* and *T. triquetra* Willd. ex Spreng. are NAD-ME-type C_4_ species, whereas *T. portulacastrum* from the second *Trianthema* clade is regarded as an NADP-ME type (Muhaidat *et al.*, 2007; Muhaidat and McKown, 2013; Koteyeva *et al.*, 2015). In the sister genus of *Sesuvium*, *Zaleya pentandra* is classified as an NAD-ME-type species and, prior to the current study, only *S. humifusum* (formerly *Cypselea humifusa* Turp.) from the American clade was known to use NADP-ME as its major decarboxylase. *Sesuvium sesuvioides* can be classified as an NADP-ME-type C_4_ based on NADP-ME being the predominant decarboxylating enzyme in its leaf tissue (Table 4). Our light microscopic studies confirm the result of Bohley *et al.* (2015) in showing that the species has a portulacelloid leaf type with centripetal positioning of the chloroplasts in the BS. These differences in biochemical subtype and leaf anatomy between and within Sesuvioideae genera argue for multiple origins of C_4_ photosynthesis in the subfamily Sesuvioideae.

To date, the use of CAM in a C_4_ species is known only from the genus *Portulaca*, where six species are able to utilize weak CAM under drought stress (Lara *et al.*, 2003; Holtum *et al.*, 2017). Based on current knowledge, Sesuvioideae does not contain strong CAM species (Sage, 2002; Veste and Thiede, 2004; Bohley *et al.*, 2015); however, only a few studies have investigated photosynthetic biochemistry and physiology in the subfamily. Martin *et al.* (1982) reported signs of nocturnal CO_2_ recycling in the halophytic annual *S. maritimum* (Walter) Britton, Sterns & Poggenb. that might be indicators for weak CAM activity. Since leaves of *S. sesuvioides* are succulent and their succulence increases with age, the existence of CAM activity was investigated in several individuals of *S. sesuvioides*. Our results showed no induction of CAM photosynthesis in unstressed individuals of the species; however, the effects of continued drought or salinity on photosynthetic metabolism have not been tested.

### Signs of photosynthetic plasticity during aging in leaves of *Sesuvium sesuvioides*

Within the lifetime of a *S. sesuvioides* leaf, the ratios of WST and airspace change dramatically. With time, abundant WST is developed and airspace is reduced. We hypothesize that this might result in an increased CO_2_ diffusion resistance caused by hydrated tissue. Succulence is thought generally to limit M conductance, increasing the transport resistance for atmospheric CO_2_ to reach photosynthetically active tissue (Maxwell *et al.*, 1997; Ripley *et al.*, 2013) and counteracting the benefits of C_4_ photosynthesis by increasing the M:BS ratio (Sage, 2002). Further studies should address whether increasing WST on the abaxial side does negatively affect CO_2_ diffusion in *S. sesuvioides* leaves. Since stomata are present on both leaf surfaces, the effects on photosynthetic performance might be negligible.

In addition to these anatomical changes, young leaves seem to have higher chlorophyll content and a slightly higher photosynthetic activity compared with the more shaded older leaves. If the changes in leaf characteristics with leaf age in *S. sesuvioides* are assumed to reflect either a division of leaf function or the gain of an additional storage function at the cost of reduced photosynthetic capacity, it might be a compromise to utilize the highly efficient C_4_ metabolism in younger leaves and store water (and probably other nutrients) in the shaded older leaves. The natural habitats of the species are sun-exposed open areas, often with saline soil, where at least temporal water limitations would be expected (Bohley *et al.*, 2017; Sukhorukov *et al.*, 2017).

In terms of enzyme activity, a pattern comparable with that of *S. sesuvioides* was reported for the C_4_ species *Portulaca oleracea* L. (Portulacaceae) by Kennedy and Laetsch (1973). Dramatic changes in metabolic characteristics between young, mature, and senescent leaves were observed in this species and apparently resulted in an increasingly C_3_-like profile in older leaves compared with younger ones.

While these findings show the influence of leaf age on C_4_ activity in an annual species, our results for *S. sesuvioides* are some of the first describing the effect of plant age on C_4_ performance in a perennial species. In addition to leaf age-related effects, it is also possible that the overall plant age has an influence on photosynthetic characteristics (see Table 1; Supplementary Table S1).

### *Sesuvium sesuvioides* appears to perform C_4_-like photosynthesis

A surprising finding in this study was the presence of RbcL in M cells as revealed by immunolabeling. The incomplete restriction of RbcL to the BS or, in other words, the weak expression of Rubisco in the M, is present in all three leaf stages (Fig. 3B, D, F). In combination with its other photosynthetic characteristics (e.g. isotope values within the C_4_ range), the RbcL immunolabeling suggests consideration should be given to categorizing *S. sesuvioides* as a C_4_-like species. The metabolism of C_4_-like plants is characterized by relatively high C_4_ cycle activity, the incomplete restriction of Rubisco to the BS, with the resulting limitations in terms of photosynthetic efficiency, and isotope and gas exchange values close to those of a fully developed C_4_ plant (Edwards and Ku, 1987).

As in the true leaves, the cotyledons of *S. sesuvioides* also exhibit labeling of RbcL in both M and BS cells (Fig. 4A, C), suggesting that there is potential for a mixed metabolism in these organs. Whereas C_4_ photosynthesis is thought to be the obligate pathway in the leaves of adult C_4_ plants, different photosynthetic biochemistry may be possible in cotyledons, as was shown for several species of Chenopodiaceae and Amaranthaceae (Wang *et al.*, 1993; Lauterbach *et al.*, 2017). Interestingly, in *S. sesuvioides*, the expression of RbcL in M cells is apparently not limited to the first few days after cotyledon emergence since the current study observed labeling in fully expanded 3-week-old cotyledons. Surprisingly, few studies have examined the photosynthetic biochemistry employed by cotyledons of C_3_–C_4_ intermediate or C_4_-like species.

While the presence of RbcL might be an indicator for a functional C_3_ cycle in the M cell type, we have no evidence that the small subunit of the enzyme is also expressed and that Rubisco holoenzyme is functional in this cell type. Leaky expression of RbcL has been reported in the leaves of young maize and *Sorghum* plants, where transcripts as well as polypeptides were observed at high levels in BS cells, but also at low levels in M cells (Meierhoff and Westhoff, 1993; Kubicki *et al.*, 1994). The results of Kubicki *et al.* (1994) hinted at a translational control of RbcL expression; however, the exact mechanism is still under investigation (Berry *et al.*, 2016).

In the C_4_-like species *Flaveria brownii* A.M.Powell that exhibits incomplete cell-specific expression of Rubisco, ~20% of the total fixed CO_2_ is directly processed in the C_3_ cycle by Rubisco in the M cells (Dias and Brüggemann, 2007). During concurrent gas exchange and carbon isotope measurements, Alonso-Cantabrana and von Caemmerer *et al.* (2016) found elevated biochemical fractionation of CO_2_ in *F. brownii* compared with the C_4_ species *F. bidentis* (L.) Kuntze. This appears consistent with our findings of more negative isotope values in *S. sesuvioides* compared with typical C_4_ values reported in the literature. Using the methodology of Alonso-Cantabrana and von Caemmerer (2016) with *S. sesuvioides* would provide data on the fraction of CO_2_ fixed by Rubisco in the M cells, giving further insights into the photosynthetic biochemistry of this species.

An additional important trait for the characterization of intermediate and C_4_-like species is the localization of glycine decarboxylase (GDC). In these photosynthetic types, GDC is restricted to the BS and enables the function of a glycine pump and subsequent recycling of CO_2_ otherwise lost due to photorespiration (Schulze *et al.*, 2016). Because of the lack of cross-reacting antibodies, we were not able to resolve the location of GDC in *S. sesuvioides* leaf tissue. Knowledge of this trait would provide additional evidence for its characterization as a C_4_-like species. Finally, prior to reaching a consensus of photosynthetic type for *S. sesuvioides*, the influence of environmental and cultivation-related factors during its growth, as well as physiological characteristics such as BS leakiness on its isotope signature, should be examined (Cernusak *et al.*, 2013).

The C_4_-like metabolism, where PEPC as well as Rubisco contributes to the total amount of fixed CO_2_, seems to be extremely rare or, to be more precise, only a few cases have been described to date. In terrestrial plants, known examples are found in the genus *Flaveria*, where three species are characterized as C_4_ like (McKown *et al.*, 2005), and are crucial representatives of a hypothetical last stage on the way towards full C_4_ in the current model of C_4_ evolution (Sage *et al.*, 2012). While being mindful of a potential hybrid origin of C_3_–C_4_ intermediate and C_4_-like species (Kadereit *et al.*, 2017), identification of species from other genera that exhibit photosynthetic biochemistries falling along the C_3_ to C_4_ continuum is important to strengthen the current view of C_4_ evolution. *Sesuvium sesuvioides* may be one such species as the results of immunolabeling, its carbon isotope values, and the CO_2_ compensation point are consistent with it employing C_4_-like metabolism. This result might not only strengthen the current model of C_4_ photosynthesis, but also supports the assumption of multiple independent origins of C_4_ photosynthesis in Sesuvioideae by adding further variation to this already photosynthetically variable subfamily. Additionally, *S. sesuvioides* exhibits photosynthetic plasticity during leaf aging that involves changes in anatomical characteristics and the activity of PEPC, the primary carboxylase of the C_4_ pathway. Consequently, this species offers unique opportunities to examine the molecular mechanisms underlying this metabolic flexibility.

## Supplementary data

Supplementary data are available at *JXB* online.

Table S1. CO_2_ compensation point measurements for *S. sesuvioides* individuals at different ages.

Table S2. Results of the comparisons of leaf age-related differences in BS, M, WST, and airspace.

Table S3. Titratable acidity of eight *S. sesuvioides* individuals at the end of the light and dark phase.

Fig. S1. Reduced phylogeny of the Sesuvioideae with anatomical leaf types and C_4_ subtype.

Fig. S2. Representative result of the control staining using antibody dilution buffer instead of a primary antibody dilution.

Fig. S3. Twenty hour measurement of the CO_2_ assimilation of an individual of *S. sesuvioides*.

Data S1. Results of the statistical analyses conducted with non-transformed data of BS percentage and chlorophyll content

Supplementary Tables S1-S3 and Figure S1-S3+Dataset S1

## Abbreviations

BS: bundle sheath
CAM: Crassulacean acid metabolism
Γ: CO_2_ compensation point
GDC: glycine decarboxylase
M: mesophyll
NAD-ME: NAD-dependent malic enzyme
NADP-ME: NADP-dependent malic enzyme
PPFD: photosynthetic photon flux density
PEPC: phospho*enol*pyruvate carboxylase
PEPCK: phospho*enol*pyruvate carboxykinase
RbcL: large subunit of Rubisco
WST: water storage tissue
