# Supplementary material for: C4-like photosynthesis and the effects of leaf senescence on C4-like physiology in Sesuvium sesuvioides (Aizoaceae)
Source: J Exp Bot. 2019 Jan 24;70(5):1553–65. doi: 10.1093/jxb/erz011 (PMC6411375; doi:10.1093/jxb/erz011)
Supplement: Supplementary Tables S1-S3 and Figure S1-S3+Dataset S1 [file erz011_suppl_supplementary_material.pdf]

Supplementary Data

Supplementary Table S1. CO_2 compensation point (Γ) measurements and maximum assimilation rate at 380 ppm CO_2 for each measurement of the 17 *Sesuvium sesuvioides* individuals included in the study. The values have been sorted by age in months at the time of measurement. A_{max} shows the maximum assimilation rate at 1400 µmol m^{-2} s^{-1} PPFD and 380 ppm CO_2 reached in an individual measurement. Mean CO_2 compensation point as well as SD have been calculated for each of these groups. Additionally, the mean and SD for all measurements taken during the summer (Apr–Sep) and winter (Oct–Mar) were calculated. The grey shaded measurement was not included into further calculations since it is a repeated measurement from the same day, but is presented to reflect the variation within an individual.

age in months	individual	dates of measurements	A_{max} [µmol m^{-2} s^{-1}]	Γ [ppm]	$Γ_{mean}$ [ppm]	SD of $Γ_{mean}$ [ppm]
8	a	18. Aug 16	6.502	0.739	0.201	1.907
	a	24. Aug 16	9.814	-2.589		
	a	24. Aug 16	9.244	5.333		
	b	22. Aug 16	7.502	0.934		
	b	24. Aug 16	7.165	1.718		
11	e	29. Aug 16	3.907	5.458	3.355	2.974
	g	23. Aug 16	5.348	1.252		
12	d	29. Sep 16	6.677	2.998	2.672	1.421
	i	29. Sep 16	7.032	1.081		
	j	30. Aug 16	4.441	4.449		
	j	26. Sep 16	6.03	2.161		
13	a	01. Feb 17	6.657	3.049	3.552	1.682
	a	06. Feb 17	3.662	1.069		
	b	01. Feb 17	2.498	3.258		
	b	07. Feb 17	3.216	5.329		
	b	17. Feb 17	4.541	3.288		
	d	26. Oct 16	4.293	6.184		
	e	24. Oct 16	5.514	0.886		
	f	26. Oct 16	4.467	4.649		
	g	26. Oct 16	5.008	3.658		
	h	25. Oct 16	4.651	5.101		
	i	26. Oct 16	4.748	2.601		
16	c	31. Jan 17	1.163	23.722	9.285	8.557
	f	23. Jan 17	2.045	2.813		
	h	24. Jan 17	3.259	0.114		
	i	24. Jan 17	2.362	1.813		
	i	30. Jan 17	2.354	5.999		
	j	25. Jan 17	3.845	5.243		
	k	12. Jul 16	3.738	7.565		
	l	04. Jul 16	0.845	20.188		

	l	14. Jul 16	1.055	16.106		
17	c	21. Feb 17	2.278	7.521	8.182	3.551
	f	13. Feb 17	2.915	6.907		
	f	21. Feb 17	2.198	7.141		
	f	28. Feb 17	2.238	3.903		
	h	20. Feb 17	3.569	6.027		
	i	06. Feb 17	3.529	5.318		
	k	19. Aug 16	1.136	17.659		
	n	18. Aug 16	2.236	11.551		
	n	19. Aug 16	3.577	7.021		
	o	25. Aug 16	2.926	9.213		
	p	25. Aug 16	5.738	9.465		
	l	26. Aug 16	2.059	5.048		
	p	29. Aug 16	3.861	9.596		
18	c	13. Mar 17	2.242	8.589	7.199	2.422
	c	15. Mar 17	1.801	5.27		
	d	13. Mar 17	2.976	7.111		
	g	15. Mar 17	1.869	6.419		
	o	07. Sep 16	6.123	5.139		
	p	07. Sep 16	4.359	5.876		
	q	15. Sep 16	6.749	11.989		
19	c	04. Apr 17	5.304	5.744	8.777	2.659
	d	10. Apr 17	5.676	9.753		
	h	11. Apr 17	1.652	12.074		
	h	19. Apr 17	2.718	6.355		
	j	10. Apr 17	3.425	9.959		
20	d	10. May 17	1.868	6.019	8.665	3.742
	j	11. May 17	1.479	11.311		
22	k	25. Jan 17	1.081	16.095	7.456	5.776
	l	23. Jan 17	2.119	4.328		
	l	30. Jan 17	3.305	5.187		
	o	24. Jan 17	2.159	4.213		
23	k	20. Feb 17	1.955	8.164	7.026	2.68
	k	28. Feb 17	2.311	3.879		
	m	01. Feb 17	3.288	7.261		
	m	10. Feb 17	2.881	9.357		
	m	17. Feb 17	3.056	10.831		
	m	21. Feb 17	3.629	8.153		
	n	13. Feb 17	5.852	2.655		
	o	06. Feb 17	2.954	10.006		
	o	17. Feb 17	2.571	7.63		
	p	20. Feb 17	4.025	4.711		
	q	23. Feb 17	3.926	4.643		
24	m	14. Mar 17	4.127	10.318	8.432	2.668
	p	14. Mar 17	4.739	6.545		
25	l	12. Apr 17	2.265	10.896	8.635	4.891
	n	11. Apr 17	7.428	8.003		

	n	18. Apr 17	10.14	-0.978		
	o	13. Apr 17	1.972	14.424		
	p	10. Apr 17	6.703	11.273		
	p	13. Apr 17	4.578	6.823		
	q	11. Apr 17	4.401	10.005		
26	k	12. May 17	1.813	7.579	7.438	3.224
	k	29. May 17	1.994	13.357		
	l	11. May 17	1.548	6.611		
	n	15. May 17	5.975	2.475		
	p	16. May 17	2.887	6.746		
	q	10. May 17	3.8	6.799		
	q	15. May 17	1.822	8.496		
winter	Oct–Mar				6.067	4.034
summer	Apr–Sep				6.939	4.436

Supplementary Table S2. Results of the multiple comparisons analyses of leaf stage related differences in bundle sheath (BS), mesophyll (M), water storage tissue (WST) and airspace. The values have been calculated using multiple comparisons with p-value adjustment following the Holm-Bonferroni method. In the analysis of BS, data raised to the power of λ has been used. y = young leaf; m = mature leaf, s = senescing leaf.

	Estimate	Standard error	z-value	p-value
BS (power-transformed data; λ = -0.3)				
y - m	0.095	0.017	5.74	< 0.001
y - s	0.153	0.017	9.24	< 0.001
m - s	-0.058	0.017	-3.36	0.001
M				
y - m	7.492	1.753	4.274	< 0.001
y - s	12.851	1.753	7.331	< 0.001
m - s	-5.359	1.819	-2.946	0.003
WST				
y - m	-19.280	1.963	-9.820	< 0.001
y - s	-30.111	1.963	-15.336	< 0.001
m - s	10.830	2.045	5.295	< 0.001
airspace				
y - m	6.524	1.134	5.755	< 0.001
y - s	10.860	1.134	9.581	< 0.001
m - s	-4.336	1.178	-3.683	< 0.001

Supplementary Table S3. Titratable acidity of eight *Sesuvium sesuvioides* individuals at the end of the light and dark period. Leaf extracts were titrated against 0.1 M NaOH.

	evening [µmol H^+ mg FW^{-1}]	morning [µmol H^+ mg FW^{-1}]	difference [µmol H^+ mg FW^{-1}]
a	12.43	14.37	1.95
b	14.87	15.21	0.34
d	11.48	10.8	-0.68
k	13.33	11.23	-2.1
l	10.71	11.97	1.26
n	13.25	15.01	1.76
o	8.54	11.19	2.65
p	13.01	14.69	1.69

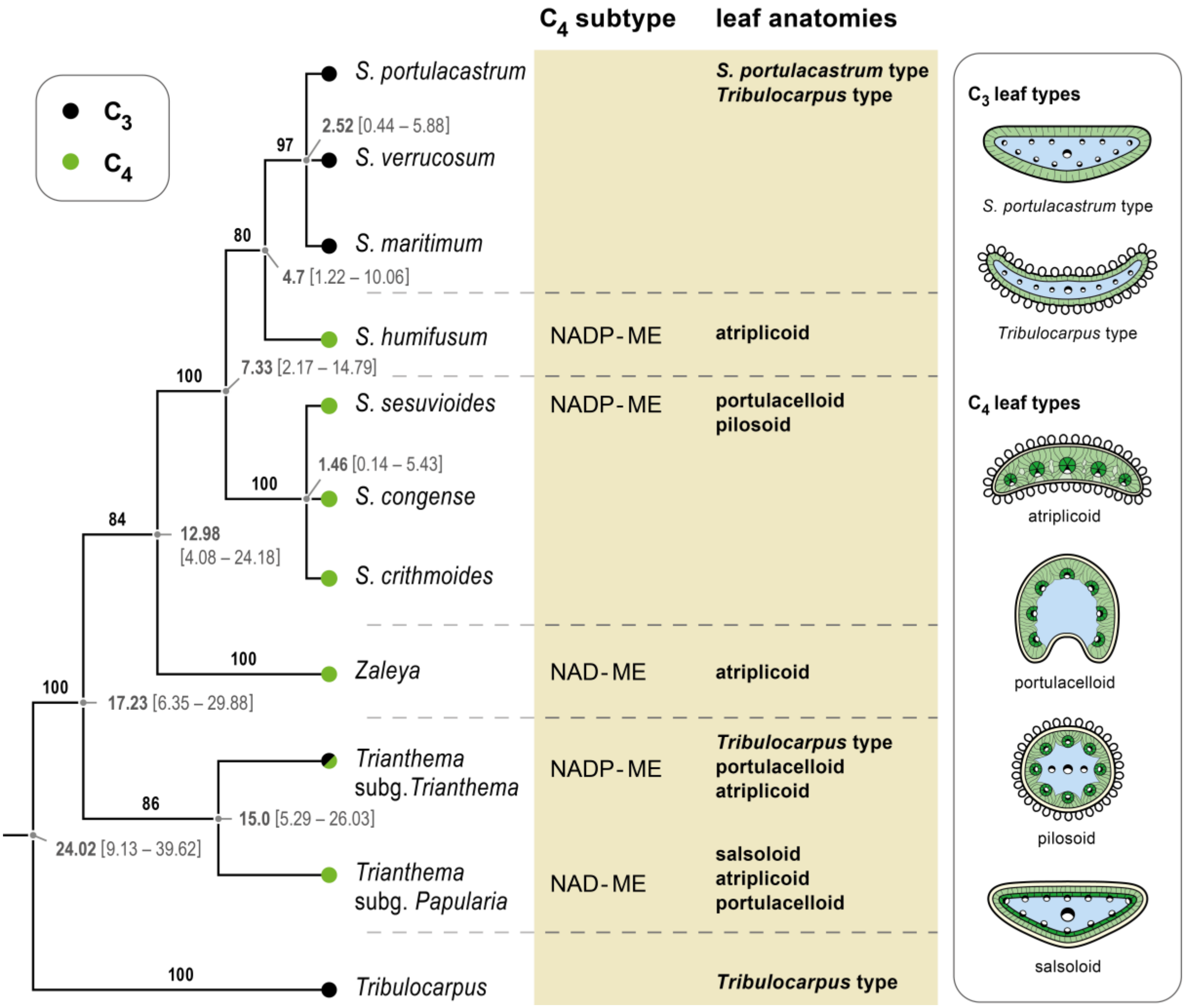

Supplementary Figure S1. Phylogeny of Sesuvioideae with focus on *Sesuvium*. *Trianthema*, *Tribulocarpus* and *Zaleya* are represented at the (sub-)generic level. Black-filled circles at terminal branches denote C_3 photosynthesis, green-filled circles show the presence of C_4 photosynthesis. C_4 subtype identity at the species level is shown only within *Sesuvium*; for the other genera, subtypes that have been identified are shown. Notation of leaf anatomies represents the presence of a type at the genus/clade level. Phylogenetic tree modified from Bohley *et al.* 2015, Perspectives in Plant Ecology, Evolution and Systematics 17, 116–130.

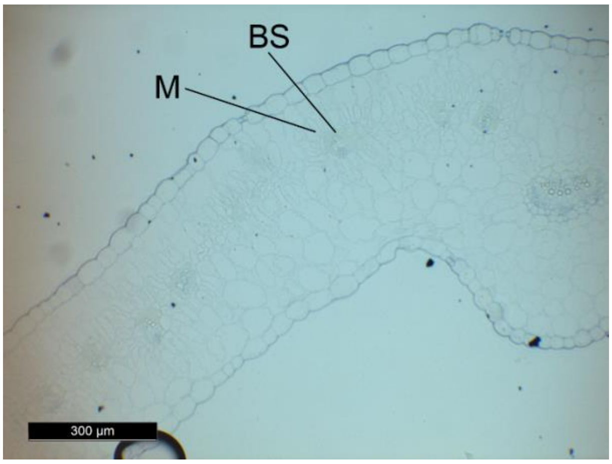

Supplementary Figure S2. Representative result of an immunolabeling control leaf section using antibody dilution buffer in place of a primary antibody. Apart from this difference, the controls were treated similar to the sections labeled using a primary antibody. No non-specific reaction labeling was detected.

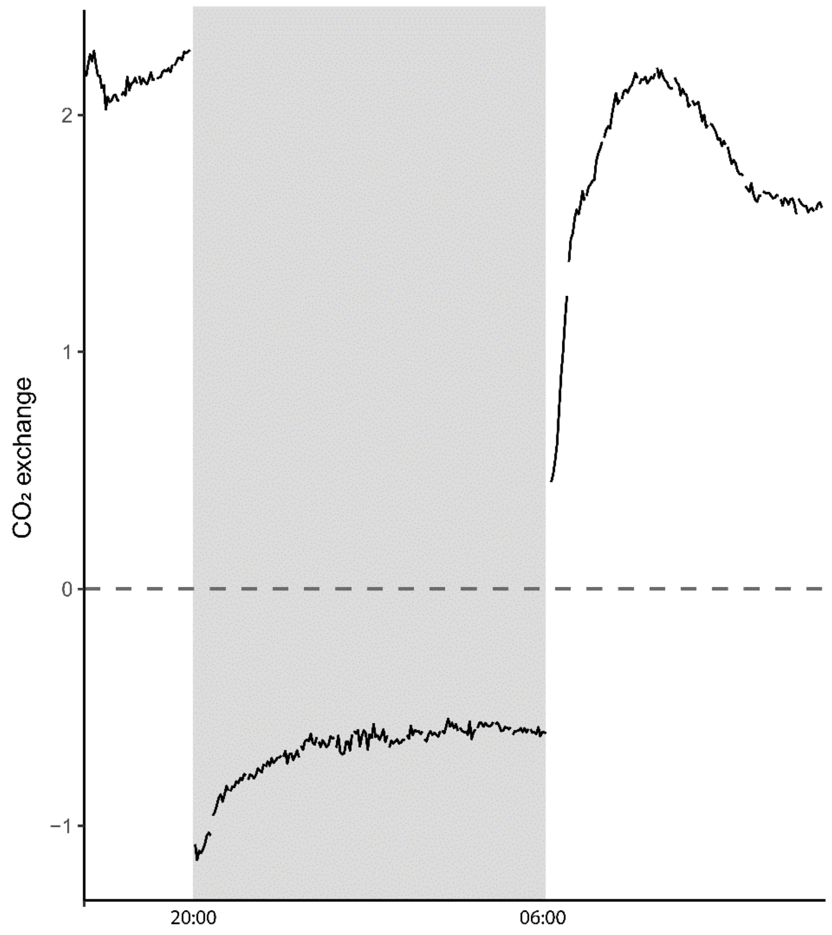

Supplementary Figure S3. Diurnal net assimilation rate of a *Sesuvium sesuvioides* individual. Measurements were performed at 380 ppm CO_2 under 14 h light/ 10 h dark cycles. Grey shading marks the dark period. The total duration of the measurement was 20 hours.

Supplementary Dataset S1. Results of the statistical analyses conducted with non-transformed data of BS percentage and chlorophyll content. The same procedure employed for the transformed data, was used with the non-transformed data to illustrate differences in the results.

The results of the analysis with transformed data of BS percentage and chlorophyll content showed a significant influence of leaf stage on the respective dependent variables (BS or chlorophyll content) and detected significant differences between all leaf stages. While the influence of leaf stage on the dependent variables (BS and chlorophyll content) was also significant when using the non-transformed data sets, the results of the multiple comparison analyses differ slightly: Significant differences were detected between young and mature leaves as well as between young and senescing leaves, but not between mature and senescing leaves in the analysis of BS percentage and also in the analysis of chlorophyll content.

Results of linear mixed effects models used to assess the effect of leaf stage on the percentage of BS and on chlorophyll content. For model selection, the BIC of the model including the effect in question (leaf stage) was compared to a model without this effect (reduced model).

	value	Standard error	df	t-value
Bundle sheath				
Reduced model (BIC = 147.9128)				
Full model (BIC = 124.0441)				
$Leaf_{young}$	11.825	1.034	14	5.161
Intercept ($Leaf_{mature}$)	6.49	0.907	14	7.158
$Leaf_{senescing}$	4.631	1.073	14	-1.733
Chlorophyll content				
Reduced model (BIC = 345.3414)				
Full model (BIC = 147.2821)				
$Leaf_{young}$	27.095	3.383	31	3.459
Intercept ($Leaf_{mature}$)	15.392	2.461	31	6.254
$Leaf_{senescing}$	8.038	4.073	31	-1.806

The results of the selected linear mixed model were summarized using F-statistics to determine the influence of the leaf stage on the dependent variables.

Bundle sheath
F-value = 26.627 p-value = < 0.0001

Chlorophyll content
F-value = 12.334 p-value = 1e-04

Results of the multiple comparisons analyses of leaf stage related differences in BS percentage and chlorophyll content. The values have been calculated using multiple comparisons with p-value adjustment following the Holm-Bonferroni method. Significant p-values are indicated by bold letters. y = young leaf; m = mature leaf, s = senescing leaf.

	Estimate	Standard error	z-value	p-value
BS				
y - m	5.335	1.034	5.161	**4.92e-07**
y - s	7.194	1.034	6.959	**1.03e-11**
m - s	-1.859	1.073	-1.732	0.0832
Chlorophyll content				
y - m	11.703	3.383	3.459	**0.00108**
y - s	19.057	4.073	4.679	**8.66e-06**
m - s	-7.354	4.073	-1.805	0.07100
